# Prostate cancer temporal and regional trends in Brazil

**DOI:** 10.32604/or.2024.052179

**Published:** 2024-09-18

**Authors:** MEHRSA JALALIZADEH, HEVELINE RAYANE MOURA ROESCH, FERNANDO KORKES, QUOC DIEN-TRINH, LEONARDO OLIVEIRA REIS

**Affiliations:** 1 UroScience, School of Medical Sciences, State University of Campinas, Campinas, 13083-872, Brazil; 2 Division of Urology, Faculdade de Medicina do ABC, São Paulo, 09051-040, Brazil; 3 Division of Urological Surgery and Center for Surgery and Public Health, Brigham and Women’s Hospital, Harvard Medical School, Boston, MA 02115, USA; 4 ImmunOncology, Pontifical Catholic University of Campinas, PUC-Campinas, Campinas, 13087-571, Brazil

**Keywords:** Prostate cancer (PCa) epidemiology, Regional disparities, Temporal trends, COVID-19 pandemic, Screening

## Abstract

**Objectives:**

The Brazilian Unified Health System (Sistema Único de Saúde−SUS) is the universal public healthcare system of Brazil that maintains a nationwide database of its patients. Our primary objective was to analyze regional and temporal trends, while our secondary goal was to establish correlations between states’ health economy status and their prostate cancer (PCa) epidemiology.

**Methods:**

We analyzed Brazil’s nationwide data on prostate cancer (PCa) incidence, mortality, and care gathered between 2013 and 2021 by the Information Technology Department of SUS (DATA-SUS), updated monthly using the International Classification of Diseases (ICD-10) code.

**Results:**

In the period, 273,933 new cases of PCa and 135,336 PCa deaths were reported in men aged 50 years or over in Brazil. The median annual PCa-specific incidence rate (PCSIR) ranged from 14.7 in the Southeast to 6.9 in the North region and the median annual PCa-specific mortality rate (PCSMR) ranged from 7.7 in the Northeast to 6.0 in the South region (per 10,000 men >50). The median annual mortality to incidence ratio (MIR) was highest in the North (0.88) and lowest in the Southeast region (0.44). There were significant regional differences in PCa treatment rates (per new cases); the Midwest region had the highest median annual surgery rate (0.63) while the North region had the highest median annual systemic therapy rate (0.75) and the lowest radiation therapy rate (0.06). Temporal analysis of the data showed significant change in annual rate trends after the year 2018 for PCSIR (coefficient [*β*] = +3.66, *p* < 0.001), any treatment (*β* = −0.06, *p* = 0.016), surgery ([SR] *β* = +0.05, *p* = 0.017) radiation therapy ([RTR] *β* = −0.06, *p* = 0.005) and systemic therapy ([STR] *β* = −0.10, *p* = 0.002). After the 2020 pandemic, annual PCSIR decreased (*β* = −2.15, *p* = 0.002) but annual PCSMR, MIR, and treatment rates remained stable. Correlation studies showed that the PCSIR was strongly negatively correlated with STR (*p* < 0.001) and positively correlated with RTR (*p* = 0.004). MIR was positively correlated with STR (*p* < 0.001) and negatively correlated with the number of robotic surgical systems per million population (*p* = 0.003).

**Conclusion:**

Our data shows that PCa care is dependent on the region and is likely influenced by access to treatment options. Furthermore, changes after the year 2018 underscore the influence of international guidelines on Brazilian clinicians’ decision-making especially concerning population screening which in turn affected incidence and treatment rates. Limitation of our study includes limited patient-related information and data on private practices as well as an unknown impact of traveling patients.

## Introduction

Cancer surveillance is key to healthcare planning and evaluation. Prostate cancer (PCa) is the third most commonly diagnosed cancer worldwide accounting for 7.3% of diagnosed cancer. In Brazil, PCa is expected to be the most frequently diagnosed cancer in men (30%) [1,2]. The human development index, national screening programs, and international guidelines on PCa screening and management are shown to significantly influence PCa-specific incidence and mortality rates (PCSIR and PCSMR) [3–5]. The COVID-19 pandemic is associated with the disruption of screening programs and biopsies, consequently altering PCSIRs and the average stage at diagnosis [6,7].

Selecting the appropriate treatment for prostate cancer (PCa) presents a complex challenge. Much of this decision-making process hinges on accurately assessing the risk of elderly patients succumbing to slow-growing cancer before other age-related diseases. *Watchful waiting* involves providing palliative care if symptoms arise and is typically reserved for individuals with a life expectancy of <10 years. On the other hand, *active surveillance* involves closely monitoring low-risk PCa in patients with a life expectancy >10 years, intervening with treatment only in case of disease progression. The objective of active surveillance is to avoid the potential adverse effects associated with aggressive treatments, such as urinary, sexual, and bowel dysfunction. For patients with intermediate and high-risk disease, the options include active surveillance in selected cases, radical prostatectomy, systemic therapy, and radiation therapy in localized cases. Systemic therapy in PCa typically involves endocrine interventions such as androgen deprivation therapy (ADT). This treatment may be administered either as a neoadjuvant to surgery or radiation, or reserved for metastatic cases [8,9]. Here, we report our analysis of data acquired from the Brazilian universal public healthcare system on PCa across Brazil between the years 2013 and 2021. Our main aim was to detect regional and temporal trends in PCa epidemiology and care.

Our secondary aim was to procure associations between these statistical indices and health economy status. We hypothesize that during this period, PCa-related care was affected by regional access to healthcare, screening programs, changes in international guidelines, and the COVID-19 pandemic.

## Materials and Methods

### Information sources

The Brazilian Unified Health System (Sistema Único de Saúde−SUS) is the universal public healthcare system of Brazil funded in the 1990s [10]. Patients enter the system through primary care; those with suspicion of cancer are referred to secondary care and confirmed cases are referred to specialized tertiary care centers such as oncology centers, oncology units, and hospitals where the diagnosis is confirmed and radiation therapy, systemic therapy (chemotherapy, hormonal therapy [androgen deprivation therapy-ADT], etc.), surgery, and palliative care is administered accordingly. Information Technology Department of SUS (DATA-SUS) maintains a nationwide database of its patients which is updated monthly and uses the International Classification of Diseases (ICD-10) code [11].

Data on general deaths as well as prostate cancer new cases, treatments, and deaths was obtained on 06/15/2023 from the following DATA-SUS warehouses: Ambulatory Information System (SIA) (sia.datasus.gov.br/principal/index.php, accessed on 25/01/2024), Hospital Information System (SIH) (sihd.datasus.gov.br/principal/index.php, accessed on 25/01/2024), and Cancer Information System (SISCAN) (www.inca.gov.br/publicacoes/manuais/manual-do-sistema-de-informacao-do-cancer-siscan-modulos-1-2-3-e-4, accessed on 25/01/2024). The information was limited to the years 2013 to 2021 and men over the age of 50. Information on population was obtained from the Brazilian Institute of Geography and Statistics (IBGE) from their population projection estimates between 2013 and 2021 (www.ibge.gov.br, accessed on 25/01/2024). Information on health expenditure was obtained from the Institute for Health Policy Studies (IEPS), a non-profit independent organization (ieps.org.br/institucional, accessed on 25/01/2024). This information was limited to the year 2021. Information on the number of SUS hospitals in the year 2023 was obtained from the database of the Brazilian Ministry of Health-The National Registry of Health Establishments–CNES (www.gov.br/saude/pt-br/acesso-a-informacao/acoes-e-programas/cebas/cnes-cadastro-nacional-de-estabelecimentos-de-saude, accessed on 25/01/2024). Information on number of robotic surgical systems per state in 2023 was obtained from www.portaldavinci.com.br(accessed on 25/01/2024). For the correlation study, these economic and healthcare indices were assumed to have been stable throughout the time of our PCa data (2013 to 2021).

Patient data was limited to the annual number of new PCa cases, PCa-related deaths, and PCa-related treatments per state, including surgeries, radiation therapies, and systemic therapies (hormonal therapy, chemotherapy, etc.). DATA-SUS maintains information on patients referred to private practices [11]. No data was acquired from practices outside of the SUS.

### Statistical analysis

Yearly PCSIR and PCSMR were calculated per 10,000 men aged 50 years or older. Mortality to incidence ratio (MIR) was calculated as a proxy for cancer-specific survival [12]. The number of each PCa treatment was divided by the number of new cases to calculate their rates. Regions were compared using a two-sided nonparametric test (Wilcoxon Rank Sum), *p*-values were Holm’s corrected, and those smaller than 0.05 after correction were considered statistically significant.

### Outlier management

We used Winsorized Pearson correlation as a more robust method of correlation analysis [13]. We used rank transformation on hospitals *per capita* to reduce the impact of one outlier that was five standard deviations higher than the average [14].

### Temporal analysis

Joinpoint analysis [15] was used to detect temporal changes and its results were used as predictors in a multivariable regression model. Eventually, we incorporated year, region, and binary indicators of pre-2018 and post-2018 as well as pre-pandemic and post-pandemic (2020) as predictors of the model [5,16].

All analyses were performed on R version 4.1.2 (2021-11-01) on RStudio platform 2022.07.1 + 554 “Spotted Wakerobin” and using package tidyverse, ggpubr, and ggstatsplot [17]. The study was exempted from ethical review since it only used public deidentified data.

## Results

**Figure 1:**
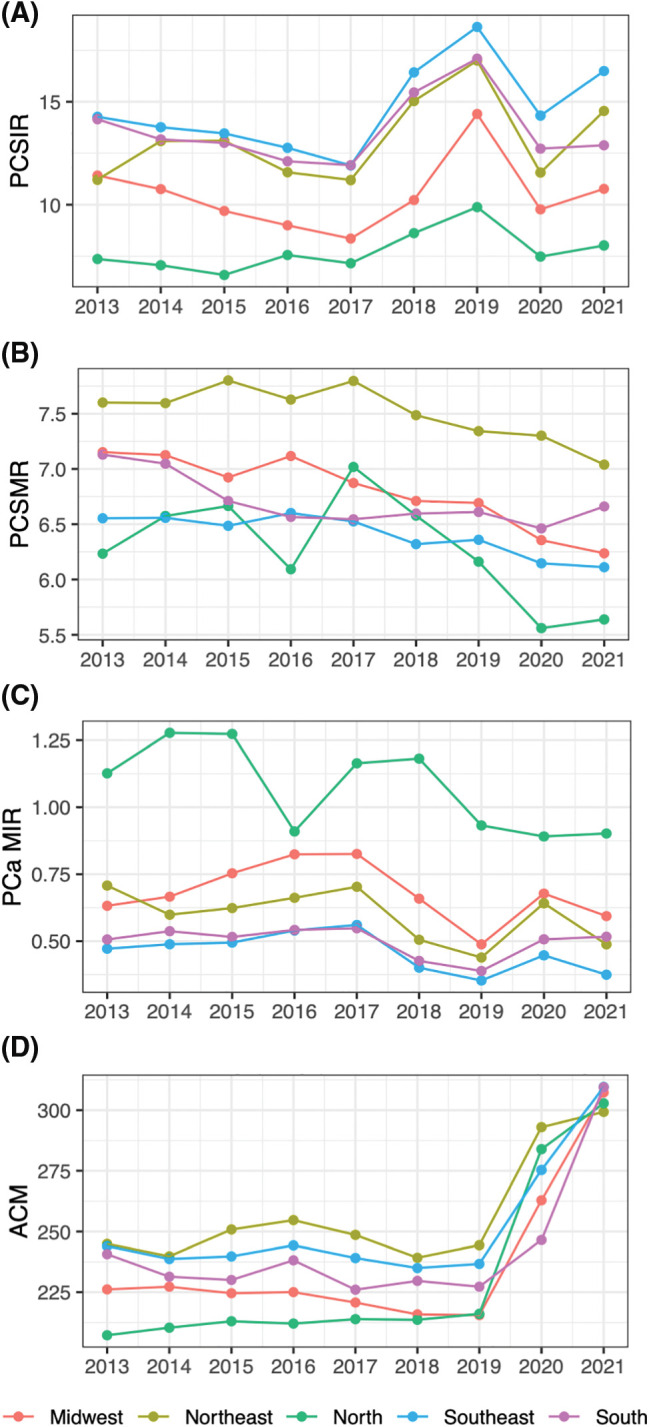
PCa statistics and ACM through years by region. (A) PCa specific incidence rate (PCSIR), (B) PCa specific mortality rate (PCSMR), (C) PCa MIR (PCSIR/PCSMR), and (D) All-cause mortality rate (ACM).

Between 2013 and 2021, 273,933 new cases of PCa and 135,336 PCa deaths were reported in men aged 50 years or over in Brazil. Table 1 summarizes median yearly rates ±IQR by region and state. The Southeast had the highest median annual PCSIR (14.7 ± 3.2) while the highest PCSMR was in the Northeast (7.7 ± 1.3). Regional differences in PCSIRs were statistically significant between all regions except South *vs*. Southeast and South *vs*. North. PCSMR was only significantly higher in the Northeast region (Fig. 1).

**Table 1:** Total PCa new cases and mortalities plus median annual rates between 2013 and 2020 per 10,000 men aged 50 years or over. Annual treatment rates are calculated by dividing their crude number by the number of new PCa cases. PCa= Prostate cancer, PCSIR = Prostate cancer specific incidence rate, PCSMR = Prostate cancer specific mortality rate. MIR= Mortality to incidence ratio, ATR = Any treatment rate (an indication of active surveillance rate), SR = Surgery rate, STR = Systemic therapy rate, RTR = Radiation therapy rate, *Median annual rate ± interquartile range (IQR)

Region/State	Total PCa cases	PCSIR*	Total PCa deaths	PCSMR*	MIR*	ATR*	SR*	STR*	RTR*
**Nationwide**	**273,933**	**11.8 ± 5.0**	**135,336**	**6.8 ± 1.7**	**0.61 ± 0.27**	**0.96 ± 0.15**	**0.11 ± 0.12**	**0.60 ± 0.19**	**0.14 ± 0.16**
**Southeast**	**138,273**	**14.7 ± 3.2**	**57,358**	**6.2 ± 1.0**	**0.44 ± 0.14**	**0.96 ± 0.16**	**0.14 ± 0.12**	**0.54 ± 0.15**	**0.24 ± 0.16**
Espírito Santo (ES)	6283	15.6 ± 1.8	2758	6.8 ± 0.4	0.43 ± 0.10	0.97 ± 0.15	0.16 ± 0.15	0.57 ± 0.15	0.14 ± 0.17
Minas Gerais (MG)	38,538	15.2 ± 3.5	13,566	5.9 ± 0.2	0.38 ± 0.09	0.97 ± 0.15	0.15 ± 0.16	0.54 ± 0.16	0.28 ± 0.17
Rio de Janeiro (RJ)	21,990	11.2 ± 2.5	13,131	6.9 ± 0.5	0.64 ± 0.10	0.96 ± 0.15	0.14 ± 0.02	0.54 ± 0.04	0.24 ± 0.13
São Paulo (SP)	71,462	15.4 ± 3.1	27,903	5.9 ± 0.1	0.38 ± 0.12	0.97 ± 0.23	0.12 ± 0.11	0.58 ± 0.20	0.22 ± 0.14
**South**	**46,018**	**13.2 ± 1.6**	**23,068**	**7.0 ± 1.7**	**0.50 ± 0.12**	**0.96 ± 0.13**	**0.12 ± 0.04**	**0.54 ± 0.1**	**0.26 ± 0.12**
Paraná (PR)	17,265	13.3 ± 0.4	8631	7.0 ± 0.5	0.53 ± 0.07	0.96 ± 0.16	0.12 ± 0.05	0.49 ± 0.05	0.32 ± 0.22
Rio Grande do Sul (RS)	18,964	13.4 ± 2.5	10,244	7.3 ± 0.5	0.55 ± 0.08	0.96 ± 0.1	0.13 ± 0.11	0.56 ± 0.08	0.24 ± 0.11
Santa Catarina (SC)	9789	12.8 ± 2.2	4193	5.6 ± 0.2	0.43 ± 0.08	0.96 ± 0.14	0.10 ± 0.05	0.60 ± 0.04	0.26 ± 0.12
**Northeast**	**65,449**	**12.4 ± 3.7**	**37,750**	**7.7 ± 1.3**	**0.61 ± 0.17**	**0.94 ± 0.21**	**0.09 ± 0.06**	**0.59 ± 0.20**	**0.15 ± 0.14**
Alagoas (AL)	2807	10.6 ± 1.7	1586	6.1 ± 0.4	0.54 ± 0.05	0.94 ± 0.04	0.05 ± 0.04	0.75 ± 0.04	0.12 ± 0.06
Bahia (BA)	19,366	12.3 ± 3.2	11,191	8.3 ± 0.3	0.64 ± 0.13	0.96 ± 0.31	0.07 ± 0.03	0.52 ± 0.15	0.33 ± 0.21
Ceará (CE)	10,584	12.3 ± 0.8	6033	7.4 ± 0.5	0.62 ± 0.06	0.98 ± 0.17	0.08 ± 0.03	0.66 ± 0.10	0.21 ± 0.06
Maranhão (MA)	5567	9.5 ± 2.6	3302	6.3 ± 0.6	0.68 ± 0.15	0.94 ± 0.27	0.10 ± 0.04	0.7 ± 0.22	0.11 ± 0.06
Paraíba (PB)	5080	12.4 ± 3.7	2825	7.7 ± 0.4	0.62 ± 0.22	0.99 ± 0.20	0.08 ± 0.04	0.67 ± 0.14	0.18 ± 0.07
Pernambuco (PE)	10,765	12.5 ± 1.9	6617	8.1 ± 0.3	0.64 ± 0.07	0.96 ± 0.20	0.09 ± 0.04	0.73 ± 0.15	0.11 ± 0.09
Piauí (PI)	3699	12.4 ± 2.3	2242	7.9 ± 0.6	0.68 ± 0.14	0.98 ± 0.22	0.17 ± 0.11	0.57 ± 0.10	0.16 ± 0.19
Rio Grande do Norte (RN)	4732	15.0 ± 1.1	2443	7.6 ± 0.9	0.52 ± 0.14	0.98 ± 0.21	0.12 ± 0.08	0.49 ± 0.08	0.32 ± 0.24
Sergipe (SE)	2849	14.7 ± 5.3	1511	8.3 ± 1.0	0.54 ± 0.23	0.82 ± 0.54	0.03 ± 0.11	0.6 ± 0.39	0.07 ± 0.05
**Midwest**	**15,008**	**10.1 ± 3.1**	**9556**	**6.8 ± 0.9**	**0.67 ± 0.25**	**0.94 ± 0.09**	**0.63 ± 0.07**	**0.63 ± 0.07**	**0.13 ± 0.13**
Distrito Federal (DF)	2310	9.8 ± 2.1	1680	7.0 ± 0.4	0.71 ± 0.26	0.93 ± 0.02	0.73 ± 0.13	0.73 ± 0.13	0.08 ± 0.02
Goiás (GO)	6166	9.6 ± 1.3	3851	6.2 ± 0.7	0.64 ± 0.13	0.99 ± 0.12	0.60 ± 0.02	0.6 ± 0.02	0.23 ± 0.20
Mato Grosso do Sul (MS)	3127	10.3 ± 5.4	1965	7.7 ± 1.1	0.80 ± 0.44	0.97 ± 0.41	0.63 ± 0.25	0.63 ± 0.25	0.18 ± 0.12
Mato Grosso (MT)	3405	11.8 ± 2.6	2060	6.4 ± 0.6	0.58 ± 0.13	0.95 ± 0.05	0.64 ± 0.03	0.64 ± 0.03	0.15 ± 0.11
**North**	**9185**	**6.9 ± 5.0**	**7604**	**6.0 ± 1.8**	**0.88 ± 0.53**	**0.96 ± 0.09**	**0.07 ± 0.11**	**0.75 ± 0.27**	**0.06 ± 0.17**
Acre (AC)	365	6.9 ± 2.3	323	6.0 ± 2.0	0.89 ± 0.44	0.98 ± 0.10	0.07 ± 0.06	0.78 ± 0.16	0.05 ± 0.15
Amazonas (AM)	1821	5.7 ± 3.3	1577	6.0 ± 0.9	1.05 ± 0.74	0.91 ± 0.22	0.06 ± 0.08	0.68 ± 0.44	0.12 ± 0.08
Amapá (AP)	377	8.0 ± 2.1	297	6.0 ± 1.4	0.83 ± 0.31	0.97 ± 0.09	0.02 ± 0.05	0.93 ± 0.09	0 ± 0
Pará (PA)	2929	4.7 ± 1.3	3180	5.1 ± 0.5	1.10 ± 0.36	0.97 ± 0.04	0.15 ± 0.04	0.63 ± 0.07	0.18 ± 0.10
Rondônia (RO)	2248	14.4 ± 3.4	935	6.1 ± 0.6	0.40 ± 0.17	0.93 ± 0.25	0.09 ± 0.07	0.54 ± 0.11	0.21 ± 0.26
Roraima (RR)	124	2.6 ± 1.8	227	5.7 ± 0.7	2.47 ± 0.58	1.00 ± 0.13	0.14 ± 0.22	0.87 ± 0.29	0 ± 0
Tocantins (TO)	1321	10.1 ± 1.7	1065	8.1 ± 0.9	0.79 ± 0.13	0.97 ± 0.05	0.04 ± 0.03	0.86 ± 0.11	0.07 ± 0.12

Between 2013 and 2021, 238,123 men underwent treatment for PCa in Brazil. Of these, 48,001 underwent surgery, 53,438 underwent radiation therapy, and 142,726 underwent systemic therapy. The North had the highest median MIR (0.88 ± 0.53) of all regions (*p* < 0.001 for all, Fig. 1C). This region also had the lowest median surgery rate (SR) and radiation therapy rate (RTR), (0.07 ± 0.11 and 0.06 ± 0.17, respectively) and the highest systemic therapy rate (STR) (0.75 ± 0.27). The Midwest had the highest SR (0.63 ± 0.07) and the second highest MIR (0.67 ± 0.25).

### Temporal analysis

**Figure 2:**
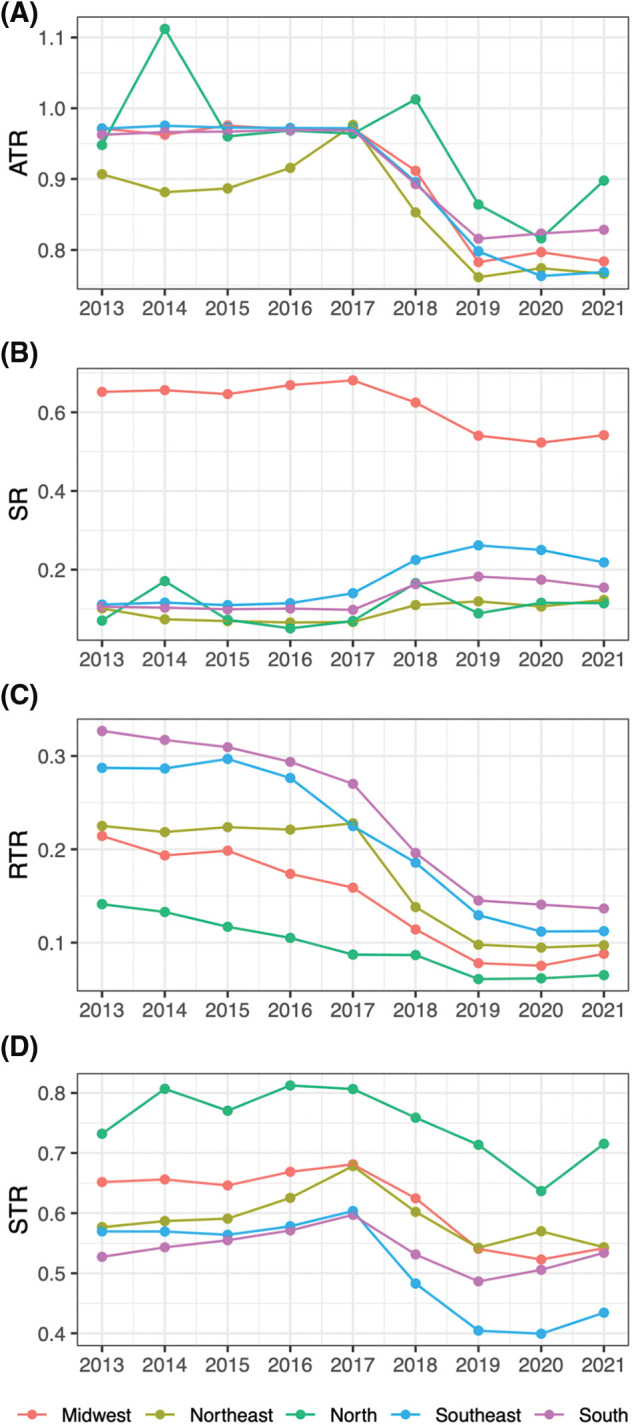
PCa treatment rates through years by region. (A) Any treatment rate (ATR), (B) surgery rate (SR), (C) radiation therapy rate (RTR), (D) systemic therapy rate (STR).

Figs. 1 and 2 spread the data through time and region to show trends. Based on the multivariable regression model (Table 2), the annual PCSIR for PCa was relatively stable between 2013 and 2018; however, after 2018 it increased (*β* = +3.66, *p* < 0.001) while after 2020 it decreased (*β* = −2.15, *p* = 0.002). In contrast, both the annual PCSMR and the MIR remained relatively unchanged throughout the studied period, extending into the pandemic years. Remarkably, all-cause mortality (ACM) experienced a significant surge during the pandemic (*β* = +58.98, *p* < 0.001).

**Table 2:** Multivariable regression model fitted to predict temporal trends in PCa statistics and treatment rates among men aged 50 years or over in different regions of Brazil. *β* = coefficient (the slope) reflects the magnitude and direction of change in the outcome variable for a unit change in the predictor, holding other variables constant. ARS = Adjusted R-Squared

	Annual change	After 2018	After 2020 pandemic	Regional difference (Midwest = reference)	Can be predicted by all variables?
PCa specific incidence rate (PCSIR)	*β* = −0.11,	*β* = +3.66,	*β* = −2.15,	Northeast: *β* = +2.14, *p* < 0.001	ARS = 0.47,
	*p* = 0.51	*p* < 0.001	*p* = 0.002	North: *β* = −2.65, *p* < 0.001	*p* < 0.0001
				Southeast: *β* = +4.18, *p* < 0.001	
				South: *β* = +3.12, *p* < 0.001	
PCa specific mortality rate (PCSMR)	*β* = +0.00,	*β* = −0.24,	*β* = −0.38,	Northeast: *β* = +0.71, *p* < 0.001	ARS = 0.24,
	*p* = 0.94	*p* = 0.32	*p* = 0.07	North: *β* = −0.52, *p* = 0.010	*p* < 0.0001
				Southeast: *β* = −0.39, *p* = 0.08	
				South: *β* = −0.10, *p* = 0.69	
PCa MIR (PCSIR/PCSMR)	*β* = −0.00,	*β* = −0.14,	*β* = +0.02,	Northeast: *β* = −0.08, *p* = 0.25	ARS = 0.29,
	*p* = 0.89	*p* = 0.15	*p* = 0.80	North: *β* = +0.39, *p* < 0.001	*p* < 0.0001
				Southeast: *β* = −0.22, *p* = 0.011	
				South: *β* = −0.18, *p* = 0.053	
All-cause mortality rate (ACM)	*β* = +1.84,	*β* = −10.18,	*β* = +58.98,	Northeast: *β* = +21.04, *p* < 0.001	ARS = 0.66,
	*p* = 0.11	*p* = 0.048	*p* < 0.001	North: *β* = −5.81, *p* = 0.16	*p* < 0.0001
				Southeast: *β* = +15.23, *p* = 0.001	
				South: *β* = +5.98, *p* = 0.23	
Any treatment (rate per new cases-ATR)	*β* = −0.00,	*β* = −0.09,	*β* = −0.05,	Northeast: *β* = −0.04, *p* = 0.13	ARS = 0.18,
	*p* = 0.87	*p* = 0.016	*p* = 0.10	North: *β* = +0.05, *p* = 0.14	*p* < 0.0001
				Southeast: *β* = −0.00, *p* = 0.90	
				South: *β* = +0.00, *p* = 0.84	
Surgery rate (SR)	*β* = −0.00,	*β* = +0.05,	*β* = −0.00,	Northeast: *β* = −0.52, *p* < 0.001	ARS = 0.81,
	*p* = 0.37	*p* = 0.017	*p* = 0.85	North: *β* = −0.51, *p* < 0.001	*p* < 0.0001
				Southeast: *β* = −0.44, *p* < 0.001	
				South: *β* = −0.48, *p* < 0.001	
Radiation therapy rate (RTR)	*β* = −0.01,	*β* = −0.06,	*β* = −0.00,	Northeast: *β* = +0.03, *p* = 0.08	ARS = 0.44,
	*p* = 0.040	*p* = 0.005	*p* = 0.85	North: *β* = −0.05, *p* = 0.004	*p* < 0.0001
				Southeast: *β* = +0.07, *p* < 0.001	
				South: *β* = +0.09, *p* < 0.001	
Systemic therapy rate (STR)	*β* = +0.01,	*β* = −0.10,	*β* = −0.06,	Northeast: *β* = −0.02, *p* = 0.37	ARS = 0.31,
	*p* = 0.10	*p* = 0.002	*p* = 0.064	North: *β* = +0.14, *p* < 0.001	*p* < 0.0001
				Southeast: *β* = −0.10, *p* = 0.002	
				South: *β* = −0.08, *p* = 0.030	

PCa treatments (Fig. 2, Table 2) remained relatively stable throughout the period, however, notable shifts emerged post-2018. Any treatment rate (ATR) decreased after this year (*β* = −0.09, *p* = 0.016), SR increased (*β* = +0.05, *p* = 0.017) while RTR and STR decreased (*β* = −0.06 and −0.10, *p* = 0.005 and 0.002, respectively).

### Correlation studies

**Figure 3:**
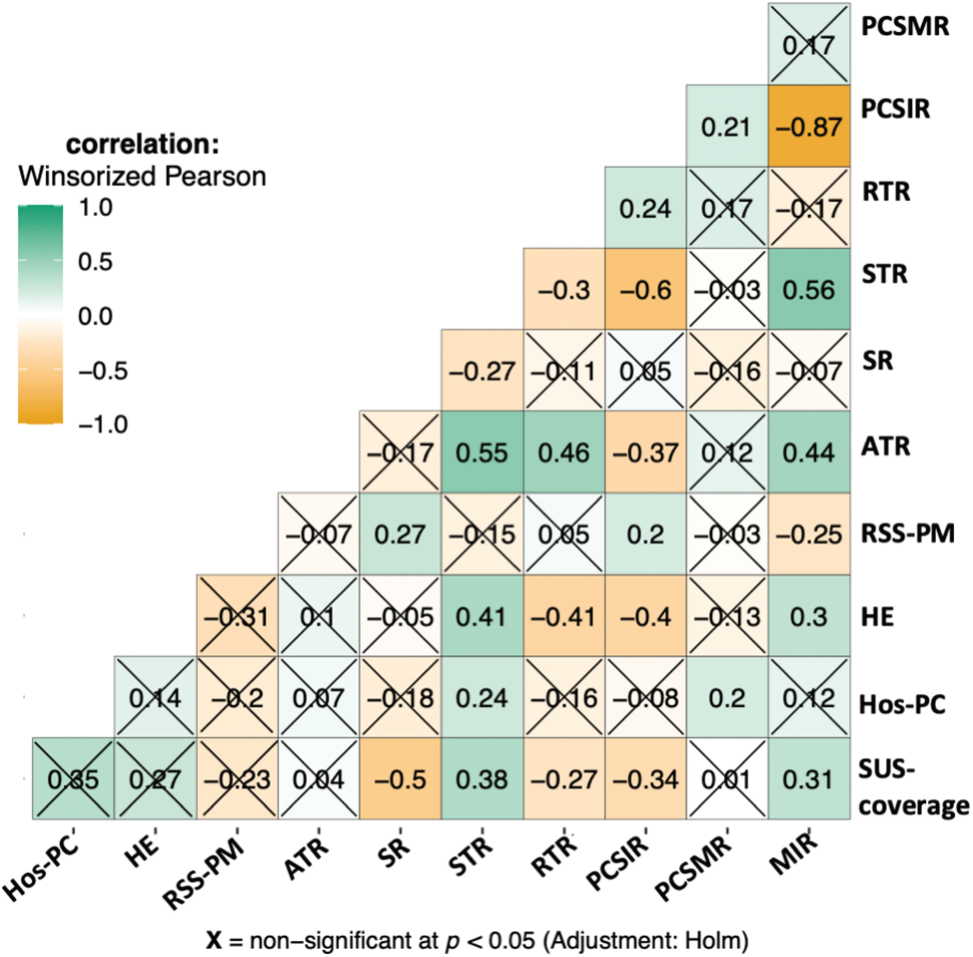
Correlogram of relationships between PCa statistics, PCa treatment rates, and health economy status of the states. STR = systemic therapy rate, SR = surgery rate, ATR = any treatment rate (indicating active surveillance/watchful waiting). The health economy status of states is represented by: SUS-coverage = percentage of hospitals covered by SUS public healthcare, Hos-PC = hospitals per capita, the state’s ranking based on the number of SUS-covered hospitals per person, HE = average health expenditure, RSS-PM = robotic surgical systems per million population.

States’ economic and healthcare status were measured based on the following indices: average health expenditure (HE), state’s ranking in the number of SUS hospitals *per capita* (Hos-PC), the number of robotic surgical systems per million population (RSS-PM), and the percentage of hospitals under SUS (SUS-coverage). Fig. 3 shows the correlation between these indices and PCSIR, PCSMR, MIR, and treatment rates. RSS-PM and SR were positively correlated (*p* < 0.001). HE was negatively correlated with PCSIR and RTR while it was positively correlated with MIR and STR (*p* < 0.001 for all). Hos-PC was positively correlated with PCSMR and STR (*p* = 0.041 and 0.004, respectively). SUS-coverage was positively correlated with MIR and STR, and negatively correlated with PCSIR, SR, and RTRs (*p* < 0.001 for all).

**Figure 4:**
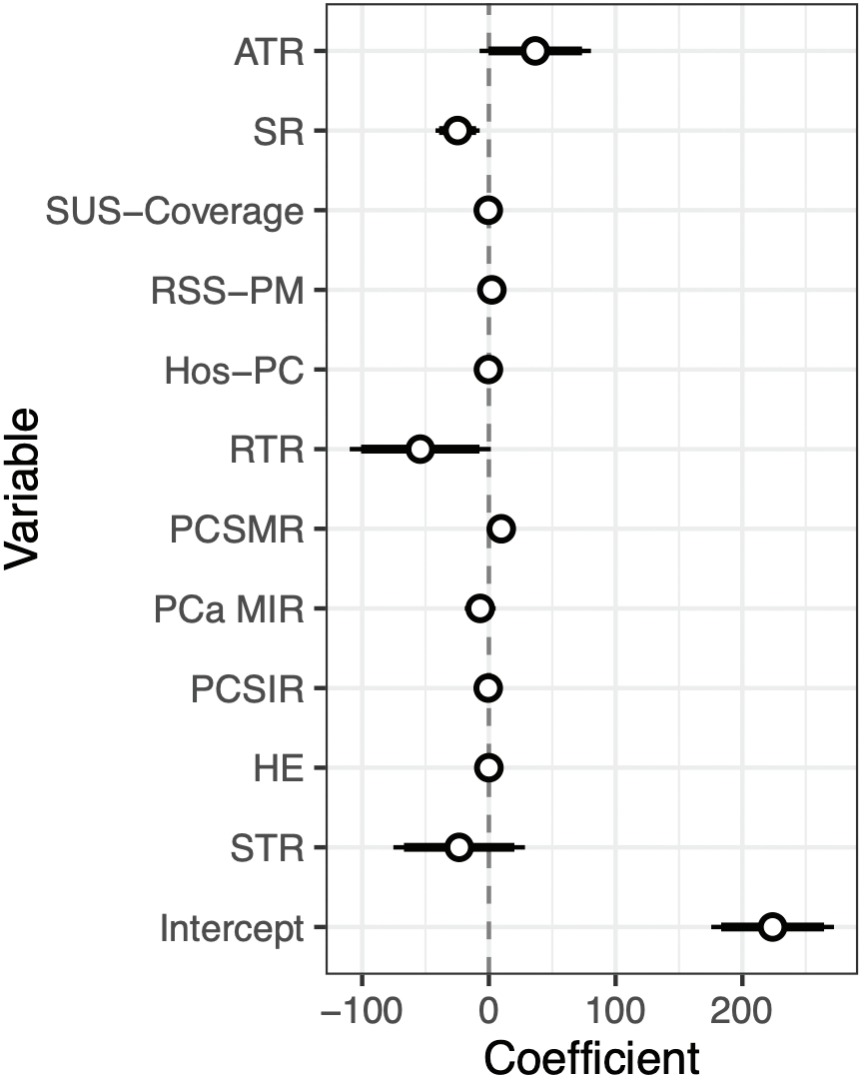
Coefficients plus 95% confidence interval of a multivariable regression model predicting all-cause mortality rate from PCa statistics and health economy status of states before the pandemic (2013–2019).

PCSIR was strongly negatively correlated with STR (*β* = −0.60, *p* < 0.001), negatively correlated with ATR (*p* < 0.001), and positively correlated with RTR (*p* = 0.004). MIR was positively correlated with ATR and STR (*p* < 0.001 for both), and negatively correlated with RSS-PM (*p* = 0.003). Fig. 4 shows a multivariable regression model predicting all-cause mortality rates before the 2020 pandemic from PCa statistics. All-cause mortality was positively correlated with PCSMR (*β* = +9.78, *p* < 0.001), and negatively correlated with SR (*β* = −24.67, *p* = 0.006). Details of states’ health economy status, coefficient estimates, as well as 95% confidence intervals, and *p*-Values pertaining to Figs. 3 and 4 are available in supplementary Tables S1–S3.

## Discussion

PCa incidence rate is largely dependent on public screening policies [3–5]. Recommendation guidelines have constantly changed in the past 13 years. In 2010 the American Cancer Society recommended screening asymptomatic men with at least 10-year life expectancy [18]. Conversely, in 2012, the U.S. Preventive Services Task Force (USPSTF) recommended against screening [19]. This recommendation was modified in 2018 to shared decision-making [20]. Most recent American and European guidelines make similar recommendations [21,22]. Two studies from the United States as well as the 2022 USPSTF evaluation concluded that the change in recommendation affected the country’s PCSMIR, PCSMR, and the incidence of metastatic disease [4,5,23].

The Brazilian health ministry in 2010 and 2014 recommended against regular screening [24,25]. Their recommendation is yet to be updated. However, in our data, we saw an increase in PCSIR in all regions after the year 2018, consistent with the 2018 change in the USPSTF recommendation. This dropped during the pandemic, consistent with the pause in screening services and biopsies during the quarantine [7]. On the contrary, neither PCSMR nor MIR were affected in these years. This is expected due to the relatively long average survival of PCa patients [26]. A 2023 study in the USA similarly found an increase in PSA screening after 2018 and a decrease after 2020 [27].

The choice of treatments significantly changed after 2018 in all regions of Brazil. Before 2018, ATR was close to 100%, indicating that almost all newly diagnosed PCa cases received some form of treatment. After this year, ATR significantly dropped (*β* = −0.09, *p* = 0.016). An augmented rate of active surveillance due to an increased number of early-stage PCa cases discovered through increased screening is a possible explanation. The rate of other treatments also changed after 2018 (Fig. 1, Table 2). Increased SR and decreased RTR/STR could either be due to an increased number of early-stage cancers or improved access to high-technology surgery as the correlogram (Fig. 3) shows a correlation between access to robotic surgery and SR.

### Regional differences

Our analysis of regional differences (Table 1, Fig. 1) is not in concordance with a prediction study by Santos et al. [2] While we saw the highest PCSIR in the Southeast region followed by the South, Santos et al.’s predicted the highest PCSIR in the Northeast followed by the Midwest. This is possibly due to their different information source (Population-Based Cancer Registries 1987–2019).

A 2022 study by Iser et al. analyzed PCSMR trends in different regions of Brazil between 1990 and 2019 [28]. Similar to our study, they found the highest PCSMR in the Northeast region. In our study, the Midwest had the second highest PCSMR, contrary to Iser et al.’s which showed the North to have the second highest mortality. This discrepancy is possibly due to the difference in the time of our data. Similar to our study, they found high PCSMRs in the states of Bahia (BA) and Tocantins (TO).

We saw a significant difference among regions in their choice of treatment. While the Midwest region treated the majority of cases with surgery (median rate 0.67), other regions preferred systemic therapy (ranging between 0.75 in the North to 0.54 median rate in the South) and radiation therapy (from 0.26 in the South to 0.06 median rate in the North, *p* < 0.001). The difference could be the result of better access to robotic surgery in the Midwest (0.57 RSS-PM, Supplementary Materials) and/or limited access to radiation therapy or systemic therapy in the region. According to Fonseca et al., patients living in the Midwest between 2017 and 2018 had to commute more than 300 km to receive radiation or systemic therapy [11].

### Correlation studies

To comprehend this section of our study, it’s imperative to bear in mind three pivotal concepts:

1. Correlation does not establish causation; rather, it signifies an association between variables [29].
2. Despite PCa being the most commonly diagnosed cancer in men, it is not the primary cause of death in many countries. Surprisingly, a systematic review encompassing over 6000 autopsies of men aged over 70 revealed cancerous cells in up to 50% of the examined prostates. This underscores that a substantial portion of PCa cases evade detection without screening [1].
3. Environmental factors are not predominantly implicated in the development of PCa [1]. Consequently, fluctuations in PCSIR primarily stem from variations in screening programs [3–5].

In our data, PCSIR and STR were strongly negatively correlated (*β* = −0.60, *p* < 0.001). Clinically this makes sense since PCSIR is increased with screening, which in turn reduces the age of the patients and the number of cases that require end-stage treatment [30]. The same logic explains the negative correlation between PCSIR and ATR (*β* = −0.37, *p* < 0.001), an indicator of active surveillance rate. Screening also increased the possibility of detecting cancer in the localized stage, hence the positive relationship between PCSIR and RTR (*β* = +0.24, *p* = 0.004).

Correlation between SUS-coverage and any of the rates can be the result of selection bias: low SUS coverage may mean losing patient information to private practice. If this is not the case, then the correlations may reflect the public healthcare policy of Brazil: SUS prefers systemic therapy (*β* = +0.38, *p* < 0.001) to surgery (*β* = −0.50, *p* < 0.001) and radiation therapy (*β* = −0.27, *p* < 0.0001), which increases MIR (*β* = +0.31, *p* < 0.001). Or it could mean that states with high SUS coverage are less likely to perform PCa screening, which lowers PCSIR (*β* = −0.34, *p* < 0.001) and the stage of PCa cases at diagnosis.

The negative correlation between PCSIR and MIR is expected since the two are not truly independent. However, their strong negative correlation (*β* = −0.87, *p* < 0.001) plus the absence of correlation between PCSMR and MIR (*β* = +0.21, *p* = 0.18) can reflect the impact of screening on PCa survival. Regular PCa screening leads to discovering cases that would not need any treatment, this may falsely give the impression that screening increases PCa survival. All-cause mortality in Fig. 4 was strongly correlated with PCSMR, showing the heavy impact of this cancer on men’s health. The negative correlation between SR and all-cause mortality could result from better access to healthcare or increased screening.

### Strengths and scientific implications

DATA-SUS is one of the largest and most comprehensive healthcare systems in the world. The trends observed in their data likely provide an accurate reflection of the genuine impact of public screening policies on PCa patients. The notable shift in PCSIR metrics post-2018 underscores the significance of international guidelines in shaping clinical practices in Brazil. Consequently, these changes influenced PCa indices, as indicated by our correlation studies, which in turn strongly influenced treatment decisions. This correlation may be attributed to alterations in patient demographics, such as age, and the stage of cancer at diagnosis.

### Limitations

A major limitation of our study is the traveling patients. The states of Roraima (RR) and Amapá (AP) from the north region show zero radiation therapy cases which is due to the absence of radiation therapy centers in their states. Fonseca et al. [11] showed that patients from these states had to travel up to 2000 km for radiation or systemic therapy. Another limitation of our study is the absence of data on age, race, and cancer stage, which could explain the correlations, and information on private practices which is important in reducing bias [26,31,32].

## Conclusion

The incidence, mortality, and treatment rates of PCa are intricately linked to regional disparities, access to healthcare, and the implementation of public screening programs, which in turn, are significantly influenced by shifts in international guidelines. Public screening initiatives and healthcare accessibility play pivotal roles in altering the age and stage at which PCa is diagnosed, consequently impacting treatment decisions for affected individuals.

Moreover, the mortality rate associated with PCa exhibits a correlation with all-cause mortality rates. This interrelation underscores the broader impact of PCa on overall mortality trends and emphasizes the importance of understanding the multifaceted dynamics between PCa outcomes and broader health indicators.

## Supplementary Materials



## Data Availability

The datasets generated and/or analyzed during the current study are available from the corresponding author on reasonable request.

## Supplementary Materials

The supplementary material is available online at https://doi.org/10.32604/or.2024.052179.
