# Supplementary material for: Prostate cancer temporal and regional trends in Brazil
Source: Oncol Res. 2024 Sep 18;32(10):1565–73. doi: 10.32604/or.2024.052179 (PMC11413836; doi:10.32604/or.2024.052179)
Supplement: Supplementary file 1 [file OncolRes-32-52179-s001.docx]

**Supplementary Table S1:** Information on the health economy status of states and regions.

HE = Health Expenditure (average)

Hos-PC = Hospitals per capita, the state’s ranking based on the number of SUS-covered hospitals per person

SUS Hospitals PC (rank) = The ranking of the state in the number of SUS hospitals per capita

SUS-coverage = Percent hospitals of the state under SUS

RSS-PM = Robotic surgical systems per million population

|  | HE | Hos-PC | SUS-coverage | RSS-PM |
| --- | --- | --- | --- | --- |
| North | **1308.13** | **5** | **84.01** | **0.02** |
| AC | 1531.67 | 3.5 | 89.67 | 0.00 |
| AM | 1031.55 | 2 | 85.28 | 0.00 |
| AP | 1650.37 | 7 | 88.54 | 0.00 |
| PA | 517.14 | 3.5 | 75.02 | 0.12 |
| RO | 996.70 | 25.5 | 77.90 | 0.00 |
| RR | 2059.13 | 17 | 91.26 | 0.00 |
| TO | 1370.32 | 12 | 80.40 | 0.00 |
| Midwest | **846.14** | **2** | **66.93** | **0.57** |
| DF | - | 25.5 | 59.21 | 1.06 |
| GO | 860.86 | 23 | 64.44 | 0.14 |
| MS | 798.06 | 12 | 68.03 | 0.00 |
| MT | 879.50 | 17 | 76.02 | 1.09 |
| Southeast | **663.47** | **3.5** | **63.96** | **0.54** |
| ES | 944.54 | 12 | 68.41 | 0.52 |
| MG | 584.61 | 12 | 68.94 | 0.05 |
| RJ | 526.07 | 20 | 60.33 | 0.44 |
| SP | 598.64 | 12 | 58.17 | 1.15 |
| Northeast | **633.18** | **1** | **78.07** | **0.30** |
| AL | 696.65 | 5 | 81.29 | 0.32 |
| BA | 527.83 | 12 | 81.36 | 0.21 |
| CE | 576.74 | 17 | 79.70 | 0.11 |
| MA | 501.72 | 27 | 86.67 | 1.48 |
| PB | 517.94 | 20 | 77.41 | 0.00 |
| PE | 822.65 | 22 | 73.29 | 0.11 |
| PI | 654.77 | 7 | 78.78 | 0.00 |
| RN | 597.34 | 24 | 68.05 | 0.00 |
| SE | 803.01 | 1 | 76.05 | 0.45 |
| South | **632.27** | **3.5** | **75.41** | **0.74** |
| PR | 598.12 | 20 | 69.83 | 0.26 |
| RS | 606.94 | 7 | 82.15 | 1.82 |
| SC | 691.75 | 12 | 74.26 | 0.13 |
