# Supplementary material for: Prostate cancer temporal and regional trends in Brazil
Source: Oncol Res. 2024 Sep 18;32(10):1565–73. doi: 10.32604/or.2024.052179 (PMC11413836; doi:10.32604/or.2024.052179)
Supplement: Supplementary file 2 [file OncolRes-32-52179-s002.docx]

**Supplementary Table S2:** Details of coefficients (estimate) and confidence intervals pertaining to **Figure 3**

Conf. = confidence interval

df = degree of freedom

n.obs = number of observations

ATR = Any treatment rate (an indication of active surveillance rate).

HE = Health Expenditure (average)

Hos-PC = Hospitals per capita, the state’s ranking based on the number of SUS-covered hospitals per person

MIR= Mortality to incidence ratio

PCa = Prostate cancer

PCSIR = Prostate cancer specific incidence rate

PCSMR = Prostate cancer specific mortality rat

RSS-PM = Robotic surgical systems per million population

RTR = Radiation therapy rate

SR = Surgery rate

STR = Systemic therapy rate

SUS-coverage = Percent hospitals of the state under SUS

| **parameter1** | **parameter2** | **estimate** | **conf.level** | **conf.low** | **conf.high** | **statistic** | **df.error** | ***p*.value** | **method** | **n.obs** |
| --- | --- | --- | --- | --- | --- | --- | --- | --- | --- | --- |
| SUS-coverage | Hos-PC | 0.350923 | 0.95 | -0.03357 | 0.644931 | 1.873777 | 25 | 1 | Winsorized Pearson correlation | 27 |
| SUS-coverage | HE | 0.266696 | 0.95 | -0.13456 | 0.592808 | 1.35564 | 24 | 1 | Winsorized Pearson correlation | 26 |
| SUS-coverage | RSS-PM | -0.22583 | 0.95 | -0.55796 | 0.168657 | -1.1591 | 25 | 1 | Winsorized Pearson correlation | 27 |
| SUS-coverage | ATR | 0.04478 | 0.95 | -0.08152 | 0.169668 | 0.695869 | 241 | 1 | Winsorized Pearson correlation | 243 |
| SUS-coverage | SR | -0.50168 | 0.95 | -0.59026 | -0.40116 | -9.00308 | 241 | 3.47E-15 | Winsorized Pearson correlation | 243 |
| SUS-coverage | STR | 0.38108 | 0.95 | 0.268093 | 0.483727 | 6.398799 | 241 | 3.64E-08 | Winsorized Pearson correlation | 243 |
| SUS-coverage | RTR | -0.2668 | 0.95 | -0.37989 | -0.14585 | -4.29763 | 241 | 0.000902 | Winsorized Pearson correlation | 243 |
| SUS-coverage | PCSIR | -0.33685 | 0.95 | -0.44388 | -0.22035 | -5.5539 | 241 | 3.09E-06 | Winsorized Pearson correlation | 243 |
| SUS-coverage | PCSMR | 0.011916 | 0.95 | -0.1141 | 0.137554 | 0.185005 | 241 | 1 | Winsorized Pearson correlation | 243 |
| SUS-coverage | MIR | 0.30588 | 0.95 | 0.187244 | 0.415722 | 4.987596 | 241 | 4.68E-05 | Winsorized Pearson correlation | 243 |
| Hos-PC | HE | 0.138061 | 0.95 | -0.26338 | 0.498741 | 0.682896 | 24 | 1 | Winsorized Pearson correlation | 26 |
| Hos-PC | RSS-PM | -0.20468 | 0.95 | -0.5425 | 0.190119 | -1.04555 | 25 | 1 | Winsorized Pearson correlation | 27 |
| Hos-PC | ATR | 0.069358 | 0.95 | -0.05698 | 0.193514 | 1.079332 | 241 | 1 | Winsorized Pearson correlation | 243 |
| Hos-PC | SR | -0.1751 | 0.95 | -0.29446 | -0.05037 | -2.76098 | 241 | 0.155156 | Winsorized Pearson correlation | 243 |
| Hos-PC | STR | 0.242693 | 0.95 | 0.12053 | 0.357616 | 3.883724 | 241 | 0.004253 | Winsorized Pearson correlation | 243 |
| Hos-PC | RTR | -0.16003 | 0.95 | -0.28023 | -0.03489 | -2.51675 | 241 | 0.2374 | Winsorized Pearson correlation | 243 |
| Hos-PC | PCSIR | -0.07649 | 0.95 | -0.2004 | 0.049839 | -1.19086 | 241 | 1 | Winsorized Pearson correlation | 243 |
| Hos-PC | PCSMR | 0.20221 | 0.95 | 0.07836 | 0.319914 | 3.205366 | 241 | 0.041344 | Winsorized Pearson correlation | 243 |
| Hos-PC | MIR | 0.124307 | 0.95 | -0.00156 | 0.246299 | 1.94485 | 241 | 0.794344 | Winsorized Pearson correlation | 243 |
| HE | RSS-PM | -0.31263 | 0.95 | -0.62437 | 0.08502 | -1.61237 | 24 | 1 | Winsorized Pearson correlation | 26 |
| HE | ATR | 0.102291 | 0.95 | -0.0263 | 0.227552 | 1.566269 | 232 | 1 | Winsorized Pearson correlation | 234 |
| HE | SR | -0.05243 | 0.95 | -0.17947 | 0.07633 | -0.79968 | 232 | 1 | Winsorized Pearson correlation | 234 |
| HE | STR | 0.411377 | 0.95 | 0.2989 | 0.512581 | 6.874541 | 232 | 2.68E-09 | Winsorized Pearson correlation | 234 |
| HE | RTR | -0.41442 | 0.95 | -0.51528 | -0.30224 | -6.93596 | 232 | 1.91E-09 | Winsorized Pearson correlation | 234 |
| HE | PCSIR | -0.40321 | 0.95 | -0.50532 | -0.28996 | -6.7112 | 232 | 6.74E-09 | Winsorized Pearson correlation | 234 |
| HE | PCSMR | -0.12929 | 0.95 | -0.25334 | -0.00106 | -1.98599 | 232 | 0.771385 | Winsorized Pearson correlation | 234 |
| HE | MIR | 0.30267 | 0.95 | 0.181468 | 0.414815 | 4.83701 | 232 | 9.13E-05 | Winsorized Pearson correlation | 234 |
| RSS-PM | ATR | -0.07226 | 0.95 | -0.19632 | 0.054075 | -1.12473 | 241 | 1 | Winsorized Pearson correlation | 243 |
| RSS-PM | SR | 0.266244 | 0.95 | 0.145266 | 0.379377 | 4.287985 | 241 | 0.000913 | Winsorized Pearson correlation | 243 |
| RSS-PM | STR | -0.15478 | 0.95 | -0.27527 | -0.02952 | -2.43222 | 241 | 0.283243 | Winsorized Pearson correlation | 243 |
| RSS-PM | RTR | 0.051023 | 0.95 | -0.0753 | 0.175739 | 0.793128 | 241 | 1 | Winsorized Pearson correlation | 243 |
| RSS-PM | PCSIR | 0.201613 | 0.95 | 0.077741 | 0.319355 | 3.195493 | 241 | 0.041344 | Winsorized Pearson correlation | 243 |
| RSS-PM | PCSMR | -0.03468 | 0.95 | -0.15982 | 0.091566 | -0.53867 | 241 | 1 | Winsorized Pearson correlation | 243 |
| RSS-PM | MIR | -0.2487 | 0.95 | -0.36317 | -0.12682 | -3.98603 | 241 | 0.00294 | Winsorized Pearson correlation | 243 |
| ATR | SR | -0.16676 | 0.95 | -0.28659 | -0.04179 | -2.62552 | 241 | 0.193312 | Winsorized Pearson correlation | 243 |
| ATR | STR | 0.55457 | 0.95 | 0.460891 | 0.636026 | 10.34593 | 241 | 2.79E-19 | Winsorized Pearson correlation | 243 |
| ATR | RTR | 0.461887 | 0.95 | 0.35678 | 0.555445 | 8.084442 | 241 | 1.52E-12 | Winsorized Pearson correlation | 243 |
| ATR | PCSIR | -0.37348 | 0.95 | -0.47691 | -0.25985 | -6.25026 | 241 | 8.12E-08 | Winsorized Pearson correlation | 243 |
| ATR | PCSMR | 0.121207 | 0.95 | -0.00471 | 0.243339 | 1.895609 | 241 | 0.828915 | Winsorized Pearson correlation | 243 |
| ATR | MIR | 0.444273 | 0.95 | 0.337286 | 0.53993 | 7.698444 | 241 | 1.74E-11 | Winsorized Pearson correlation | 243 |
| SR | STR | -0.27027 | 0.95 | -0.38309 | -0.14952 | -4.35797 | 241 | 0.000719 | Winsorized Pearson correlation | 243 |
| SR | RTR | -0.11486 | 0.95 | -0.23727 | 0.011147 | -1.79496 | 241 | 0.960865 | Winsorized Pearson correlation | 243 |
| SR | PCSIR | 0.052064 | 0.95 | -0.07427 | 0.17675 | 0.809348 | 241 | 1 | Winsorized Pearson correlation | 243 |
| SR | PCSMR | -0.16375 | 0.95 | -0.28375 | -0.0387 | -2.57688 | 241 | 0.211314 | Winsorized Pearson correlation | 243 |
| SR | MIR | -0.07032 | 0.95 | -0.19445 | 0.056018 | -1.09441 | 241 | 1 | Winsorized Pearson correlation | 243 |
| STR | RTR | -0.30243 | 0.95 | -0.41257 | -0.18357 | -4.92567 | 241 | 6.09E-05 | Winsorized Pearson correlation | 243 |
| STR | PCSIR | -0.60293 | 0.95 | -0.67738 | -0.51626 | -11.7323 | 241 | 1.04E-23 | Winsorized Pearson correlation | 243 |
| STR | PCSMR | -0.03413 | 0.95 | -0.15929 | 0.09211 | -0.53015 | 241 | 1 | Winsorized Pearson correlation | 243 |
| STR | MIR | 0.564013 | 0.95 | 0.471645 | 0.644138 | 10.60329 | 241 | 4.39E-20 | Winsorized Pearson correlation | 243 |
| RTR | PCSIR | 0.241143 | 0.95 | 0.118907 | 0.356179 | 3.857387 | 241 | 0.004561 | Winsorized Pearson correlation | 243 |
| RTR | PCSMR | 0.174621 | 0.95 | 0.049873 | 0.294005 | 2.753153 | 241 | 0.155156 | Winsorized Pearson correlation | 243 |
| RTR | MIR | -0.17218 | 0.95 | -0.2917 | -0.04736 | -2.71346 | 241 | 0.164199 | Winsorized Pearson correlation | 243 |
| PCSIR | PCSMR | 0.210324 | 0.95 | 0.086776 | 0.3275 | 3.339804 | 241 | 0.02817 | Winsorized Pearson correlation | 243 |
| PCSIR | MIR | -0.87085 | 0.95 | -0.89826 | -0.8367 | -27.5037 | 241 | 1.5E-74 | Winsorized Pearson correlation | 243 |
| PCSMR | MIR | 0.169793 | 0.95 | 0.044908 | 0.289452 | 2.674732 | 241 | 0.175803 | Winsorized Pearson correlation | 243 |
