# Supplementary material for: Prostate cancer temporal and regional trends in Brazil
Source: Oncol Res. 2024 Sep 18;32(10):1565–73. doi: 10.32604/or.2024.052179 (PMC11413836; doi:10.32604/or.2024.052179)
Supplement: Supplementary file 3 [file OncolRes-32-52179-s003.docx]

**Supplementary Table S3:** Details of coefficients (estimate) and confidence intervals pertaining to **Figure 4.** This is a multivariable regression model fitted to predict all-cause mortality rates before the pandemic (2013-2019).

Conf. = confidence interval

df = degree of freedom

n.obs = number of observations

ATR = Any treatment rate (an indication of active surveillance rate).

HE = Health Expenditure (average)

Hos-PC = Hospitals per capita, the state’s ranking based on the number of SUS-covered hospitals per person

MIR= Mortality to incidence ratio

PCa = Prostate cancer

PCSIR = Prostate cancer specific incidence rate

PCSMR = Prostate cancer specific mortality rat

RSS-PM = Robotic surgical systems per million population

RTR = Radiation therapy rate

SR = Surgery rate

STR = Systemic therapy rate

SUS-coverage = Percent hospitals of the state under SUS

|  | Estimate | Std. Error | *t* value | Pr (>\|*t*\|) |
| --- | --- | --- | --- | --- |
| (Intercept) | 223.878236 | 24.691141 | 9.067 | 2.90e-16 |
| SUS_coverage | -0.461795 | 0.227597 | -2.029 | 0.04402 |
| Hos_PC. | -0.358559 | 0.198430 | -1.807 | 0.07253 |
| HE. | -0.017128 | 0.005178 | -3.308 | 0.00115 |
| RSS-PM | 2.329047 | 2.736844 | 0.851 | 0.39597 |
| STR | 36.609636 | 22.419132 | 1.633 | 0.10433 |
| SR | -24.671340 | 8.888581 | -2.776 | 0.00613 |
| STR | -23.446830 | 26.495683 | -0.885 | 0.37744 |
| RTR | -54.114296 | 28.399376 | -1.905 | 0.05841 |
| PCSIR | -0.529465 | 0.653583 | -0.810 | 0.41902 |
| PSMIR | 9.780618 | 1.320514 | 7.407 | 5.76e-12 |
| MIR | -6.852081 | 6.201212 | -1.105 | 0.27074 |
| Residual standard error: 16.31 on 170 degrees of freedom  (7 observations deleted due to missingness)  Multiple R-squared: 0.4523, Adjusted R-squared: 0.4169  F-statistic: 12.76 on 11 and 170 DF, *p*-value: < 2.2e-16 | | | | |
